# Birth cohort differences in height, weight and BMI among Indian women aged 15–30 years: analyses based on three cross-sectional surveys

**DOI:** 10.1017/S1368980021005012

**Published:** 2022-12

**Authors:** Swapna Deshpande, Tarja I Kinnunen, Sangita Kulathinal

**Affiliations:** 1 Unit of Health Sciences, Faculty of Social Sciences, Tampere University, P.O. Box 100, FI-33014 Tampere, Finland; 2 Department of Mathematics and Statistics, University of Helsinki, Helsinki, Finland

**Keywords:** Height and weight, Long-term trend, Birth cohort, Young Indian women, Multivariate regression

## Abstract

**Objective:**

To explore long-term trends in height, weight and BMI across birth cohorts among Indian women aged 15–30 years.

**Design:**

Nationally representative cross-sectional surveys.

**Setting:**

Data from three National Family Health Surveys were conducted in 1998–1999, 2005–2006 and 2015–2016. Height and weight were modelled jointly, employing a multivariate regression model with age and birth cohorts as explanatory variables. The largest birth cohort (born 1988–1992) was the reference cohort. Stratified analyses by place of residence and by marital status and dichotomised parity were also performed.

**Participants:**

437 753 non-pregnant women aged 15–30 years.

**Results:**

The rate of increase in height, weight and BMI differed across birth cohorts. The rate of increase was much lower for height than weight, which was reflected in an increasing trend in BMI across all birth cohorts. In the stratified analyses, increase in height was found to be similar across urban and rural areas. Rural women born in the latest birth cohort (1998–2001) were lighter, whereas urban women were heavier compared to the reference cohort. A relatively larger increase in regression coefficients was observed among women born between 1978 and 1982 compared to women born between 1973 and 1977 when considering unmarried and nulliparous ever-married women and, one cohort later (1983–1987 *v*. 1978–1982), among parous ever-married women.

**Conclusion:**

As the rate of increase was much larger for weight than for height, increasing trends in BMI were observed across the birth cohorts. Thus, cohort effects show an important contributory role in explaining increasing trends in BMI among young Indian women.

Height and weight are proxy indicators of nutritional wellbeing. Deficit in height-for-age indicates chronic malnutrition, and deficit or excess weight-for-height indicates acute or recent malnutrition^(1)^. Height is conceptualised as a heritable human trait influenced by environment, sociodemographic and economic determinants throughout life course, and weight is a result of the balance of energy intake and expenditure^(2–4)^. Both height and weight influence the BMI and reflect the long-term health and nutritional experience of an individual or a population.

The mean height has increased over the past century in the European continent and Central Asia^(5)^. It has been stagnant or even decreased in many African countries and in some of the South Asian countries^(6)^. An increasing trend in the mean BMI has been reported globally from 1975 to 2016^(7)^. Mimadi *et al.* reported that the younger women in the age group of 20–29 years were slightly taller than the older women above 30 years of age, based on the National Family Health Survey (NFHS) conducted in India in 2005–2006^(8)^. The mean BMI, as well as the prevalence of overweight and obesity, have been increasing in India as reported by the NFHS conducted in 1998–1999, 2005–2006 and 2015–2016^(9)^. Despite this increase, India is still grappling with undernutrition, posing a dual burden on health system.

Approximately 250 million (about 50 % of all) Indian women are 15–49 years old^(10)^. More than 40 % of them are either underweight or overweight^(11)^. Extreme body weight increases the risks of infertility and adverse pregnancy outcomes, such as preterm births, gestational diabetes, pre-eclampsia, caesarean section or fetal death^(12)^. In addition, Indians have a higher risk of non-communicable diseases at a lower BMI level compared to White population^(13)^. Hence, it is important to understand trends in height, weight and BMI among young Indian women over the past few decades. Previous studies from India have reported trends in height and BMI in a specified age group at one point in time or over short periods. These trends have been predominantly studied by presenting categorical data on the prevalence of stunting, underweight, overweight or obesity^(8,9,14)^.

There is diversity in peoples’ lifestyle, diet, occupation and physical activity levels between rural and urban regions of India. The prevalence of overweight has increased more in urban areas than in rural areas^(11)^. About 34 % of the Indian population lived in urban areas in 2019^(15)^. Epidemiological transition has impacted diet and physical activity, which are the main driving factors of height and weight. Marriage is still a universal phenomenon in India and most births are within marriages^(11,16,17)^. Married Indians have a higher BMI on average compared to their unmarried counterparts, based on the results of a large-scale survey across India in 2019^(18)^. Parity is associated with weight change, although the association is complex^(19)^. Parity and marital status are associated with the prevalence of both underweight and overweight^(20,21)^.

This paper aims to assess trends in height and weight across birth cohorts among Indian women aged between 15 and 30 years. Further, by modelling height and weight jointly, the aim is also to compare trends in height and weight with trends in BMI. We also aim to explore the trends in height, weight and BMI across birth cohorts separately according to the place of residence (urban or rural) and by marital status and dichotomised parity (nulliparous or parous) in the above age group.

## Data and methods

### Data sources

This study used publicly available data from three rounds of the nationally representative cross-sectional NFHS conducted in 1998–1999 (NFHS-2), 2005–2006 (NFHS-3) and 2015–2016 (NFHS-4). All rounds collected data on women aged 15–49 years with support from the Government of India, Ministry of Health and Family Welfare. NFHS-2 surveyed ever-married women, whereas NFHS-3 and −4 additionally included unmarried women. Detailed methods of sampling, data collection, sample size estimation and survey findings are reported elsewhere^(11,16,17)^.

The NFHS data provided information on age, birth year, place of residence, history of life events such as marriage, childbirth and number of children ever born (parity), all of which were self-reported in the interview. Women who had not ever given birth were categorised as nulliparous, and those who had given birth once or more were categorised as parous women. We refer to this dichotomised parity as ‘parity’ throughout the manuscript. Height and weight were objectively measured during the surveys by following standard protocols. Sampling weights are provided for each participant in each survey data set.

### Study participants

**Fig. 1:**
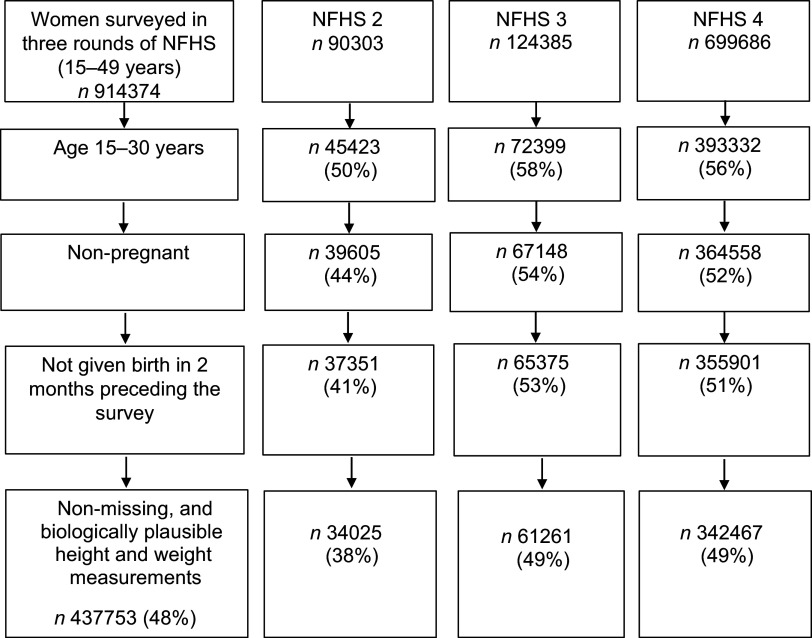
Selection of study sample. Criteria for selecting study sample of women aged 15–30 years from the Indian National Family Health Survey (NFHS) −2, −3 and −4. *n* (%) gives the number (percentage) of women satisfying the inclusion criteria for each survey

According to the reports of the three NFHS, the median age of the first marriage ranged from 17 to 19 years and the median age of women at the first birth ranged from 19·5 to 21·0 years during NFHS 2, 3 and 4. More than 95 % of ever-married women were married before the age of 30 years^(11,16,17)^. Ever-married women included currently married, widowed, separated and divorced women. Unmarried women were mainly nulliparous but included a small group of women who had children (*n* 340, 0·17 % of all unmarried women). We excluded pregnant women and women who had given birth in the 2 months preceding the survey. The study participants thus comprised of 437 753 unmarried and ever-married non-pregnant women aged 15–30 years (Fig. 1).

### Variables

**Fig. 2:**
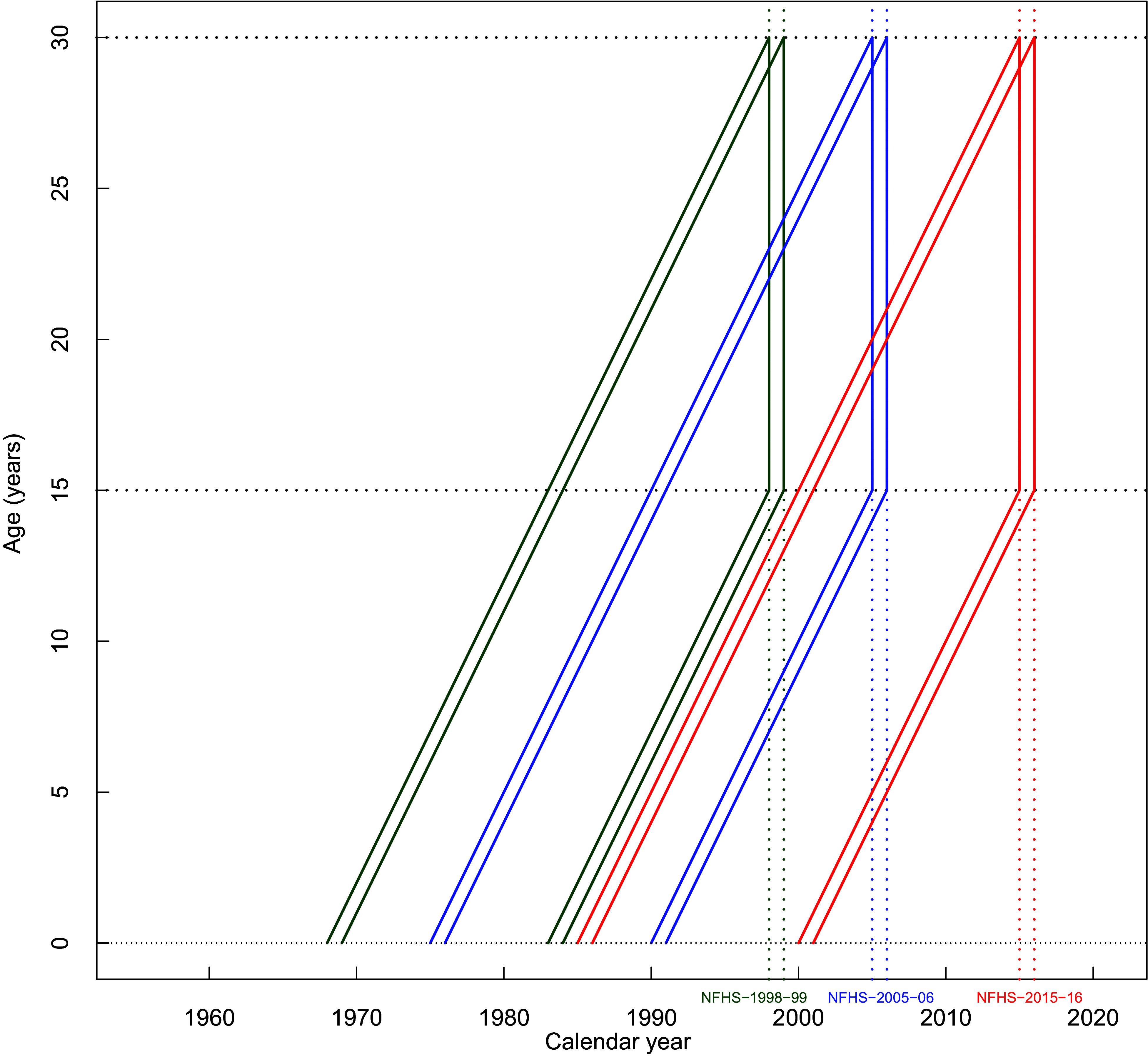
Lexis diagram. Birth cohorts spanned by the three National Family Health Surveys (NFHS) among Indian women aged 15–30 years. The diagonal lines represent birth cohorts and vertical lines represent the survey year. The area covered between the horizontal lines at 15 and 30 on the age axis defines the eligible age group. Each survey is presented by an area spanned by the birth year and age between 15 and 30 years. Green, blue and red lines indicate NFHS-2 (1998–1999), −3 (2005–2006) and −4 (2015–2016) surveys, respectively

The main explanatory variables of interest were age at the time of the survey and birth cohort. Age was measured in 1-year increments. The surveys spanned the birth cohorts of women born between 1968 and 2001 as depicted in the Lexis diagram (Fig. 2). The diagram runs from birth to the age at the time of the survey. The diagonal lines represent birth cohorts and vertical lines represent the time of the survey. Each survey is presented by an area spanned by the birth year and age between 15 and 30 years. The area covered between the horizontal lines at 15 and 30 on the age axis defines the eligible age group. Birth years were categorised by intervals of 5 years (1968–1972, 1973–1977, 1978–1982, 1983–1987, 1988–1992, 1993–1997 and 1998–2001) and used as birth cohorts in the analysis.

The main outcome variables were height (cm), weight (kg) and BMI (kg/m^2^). After excluding missing data and biologically implausible values (below 0·5^th^ and above 99·5^th^ observed percentiles) for height and weight, BMI (kg/m^2^) was calculated as the ratio of the weight in kg to the squared height in metres. The trend analyses were separately carried out in: (a) two strata defined by the place of residence (urban and rural) and (b) three strata defined by marital status and parity (unmarried, ever-married nulliparous and ever-married parous).

### Statistical analysis

Descriptive statistics were presented using mean and standard deviation for continuous variables and frequencies and percentage for categorical variables by incorporating sampling weights to account for unequal selection probabilities. Weighted mean of height, weight and BMI for a given birth cohort and age were presented using line plots.

Age effects represent accumulated physiological changes associated with the process of ageing. Birth cohort effects or generation effects are generally conceptualised as variation in the risk of a health outcome according to the year of birth^(22)^. This study attempted to test the objectives in light of age and birth cohort effect.

### Multivariate regression model

An individual’s height and weight are known to be highly correlated, and hence they were modelled jointly through their covariance structure in multivariate regression analysis using birth cohort and age as explanatory variables to explore trends in height and weight^(23)^. The multivariate regression model used is described as follows. Let *Y* be the n x m matrix of m outcomes from n individuals. The m outcomes are regressed over k covariates using the following multivariate linear regression:

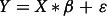




In above model, *X* is a design matrix of covariates; the first column is a vector of ones and the remaining columns correspond to k covariates. *β* is a matrix of regression coefficients and *ϵ* is an error matrix. Expectation of error is assumed to be 0. Hence, for the *j* ^*th*^ response E(*Y*_(*j*)_) = *X β*_(*j*)_. The m measurements on *i* ^*th*^ individual are assumed to be correlated and have variance–covariance matrix Σ_(m*m)_. Parameters to be estimated are regression parameter matrix *β* and common variance–covariance matrix Σ under the assumption that the individuals are independent.

For regression analysis, log-transformed height, weight and BMI were used as outcome variables because no violation of normality assumption was observed when assessed using Q-Q plots. Since the bivariate normal distribution is characterised by mean and variance–covariance matrix, the trend in the distributions of height, weight and BMI by age and birth cohorts was checked by plotting the empirical densities (results not shown). The spread of the distributions was similar, but there was a horizontal shift across the cohorts. Based on these observations, the means of the distributions were modelled jointly as a function of age and birth cohort, and assuming variance–covariance matrix constant.

In the present context, m = 2 and the outcomes were log-transformed height and weight measurements. The logarithm of BMI, which is *log (weight) – 2 log (height)*, being a linear function of log(weight) and log(height), was analysed using their bivariate regression model. Each model included age and seven categories of birth cohorts as covariates. Location of age was shifted by 15 years to give clear interpretation to the intercept. The birth cohort with the highest number of participants (1988–1992) was considered as the reference cohort in the analyses. The equality of mean height and weight across birth cohorts was tested using Pillai-Barlett’s trace test statistic for multivariate analysis of variance at a 5 % level of significance. This test is recommended as protection against nonnormality and heterogeneity of covariance matrices, and as the most robust of the multivariate ANOVA tests, with adequate power to detect true differences in a variety of situations^(24)^.

Additionally, to explore the trends in height, weight and BMI in certain subgroups, the analyses were stratified by: (a) the place of residence (urban or rural) and (b) marital status combined with dichotomised parity status (unmarried, ever-married nulliparous or ever-married parous women). Marital status and parity stratified analyses were also adjusted for the place of residence to account for inherent difference between urban and rural areas.

### Subgroup analyses

We performed two kinds of subgroup analyses. As long-term trends of changes in height, weight and BMI could be age-dependent, we checked if different age groups showed different kinds of trends and stratified the analyses, including the overall sample, by age group (15–19 years, *n* 141 318, and 20–30 years old, *n* 296 435). The social status of widowed, divorced and separated women is different than that of currently married women^(25,26)^. The change in marital status of these women, and the related changes in their life situation, might therefore have affected their body weight. Parous unmarried women are also in a more vulnerable position than married women. Hence, subgroup analyses among nulliparous unmarried women, nulliparous currently married women and parous currently married women were performed. We excluded separated, divorced, widowed and parous unmarried women from these analyses and tested the robustness of the results.

Statistical analyses were performed using *survey* and *car* packages in R (version 4.0).

## Results

### Characteristics of the study sample

The study sample from the three surveys comprised of 43 375 women, viz 34 025 (7·8 %), 61 261 (14·0 %) and 342 467 (78·2 %) from NFHS-2, −3 and −4, respectively. The average age of the women was 22 (sd 4·7) years and 69 % of the women were rural residents. Forty-three per cent of the women were unmarried, and the rest were ever-married women. Ever-married women comprised 97 % currently married women, the rest being either widowed, divorced or separated. Of the unmarried women, 0·17 % were parous women.

Table 1 represents the distribution of age, place of residence, marital status and parity by birth cohorts. The 1968–1972 birth cohort consisted of women aged 25–30 years, the second birth cohort (1973–1977) of women aged 20–30 years and the last birth cohort (1998–2001) of women aged 15–18 years. Only the 1983–1987 birth cohort had data of women ranging between 15 and 30 years. The percentage of urban women ranged from 27 % to 38 % across the birth cohorts. The first birth cohort included no unmarried women, as this cohort was contributed by the NFHS-2. The last birth cohort consisted of 96 % unmarried women and 1 % ever-married parous women. Average age at first marriage ranged between 17·4 and 18·5 years for all birth cohorts, with the exception of the last cohort since those women were younger (15–18 years). The percentage of women with more than five children reduced gradually and the percentage of women with 1–2 children increased steeply from the earlier to the later birth cohorts.

**Table 1:** Summary statistics of age, place of residence, current marital status and parity by birth cohort*

Birth cohort years	Age			Place of residence				Marital status						Parous ever-married women					
				Urban		Rural		Unmarried		Nulliparous ever-married		Parous ever-married		Parity 1–2		Parity 3–4		Parity 5+	
	Mean	sd	Min, max	*n*	%	*n*	%	*n*	%	*n*	%	*n*	%	*n*	%	*n*	%	*n*	%
1968–1972 (*n* 14 061)	28·2	1·4	25, 30	4327	32	9534	68	0		674	5	13 387	95	5588	42	5957	44	1842	14
1973–1977 (*n* 22 403)	26·0	3·1	20, 30	7949	36	14 454	64	673	3	2036	9	19 694	88	11 594	59	6744	34	1356	7
1978–1982 (*n* 24 446)	23·5	3·3	16, 28	9348	38	15 098	62	3188	13	4223	17	17 035	70	11 996	70	4444	26	595	4
1983–1987 (*n* 84 160)	26·9	4·0	15, 30	27 660	33	56 500	67	14 424	18	6094	7	63 642	75	39 180	62	21 373	33	3089	5
1988–1992 (*n* 116 023)	23·8	3·2	15, 28	35 803	31	80 220	69	33 753	29	10 321	9	71 949	62	55 078	77	15 953	22	918	1
1993–1997 (*n* 106 645)	19·9	1·6	17, 23	30 078	28	76 567	72	66 703	62	14 480	14	25 462	24	24 097	95	1349	5	16	0·1
1998–2001 (*n* 70 015)	16·0	0·9	15, 18	18 577	27	51 438	73	67 198	96	2217	3	600	1	594	99	6	1	0	

* *n* (%) are unweighted frequencies and percentage unless otherwise specified.

Percentages are based on the total number of women in each birth cohort except for parity among ever-married parous women. Those percentages are based on the number of parous ever-married women in the respective birth cohort.

### Mean height, weight and BMI by age and birth cohorts

**Fig. 3:**
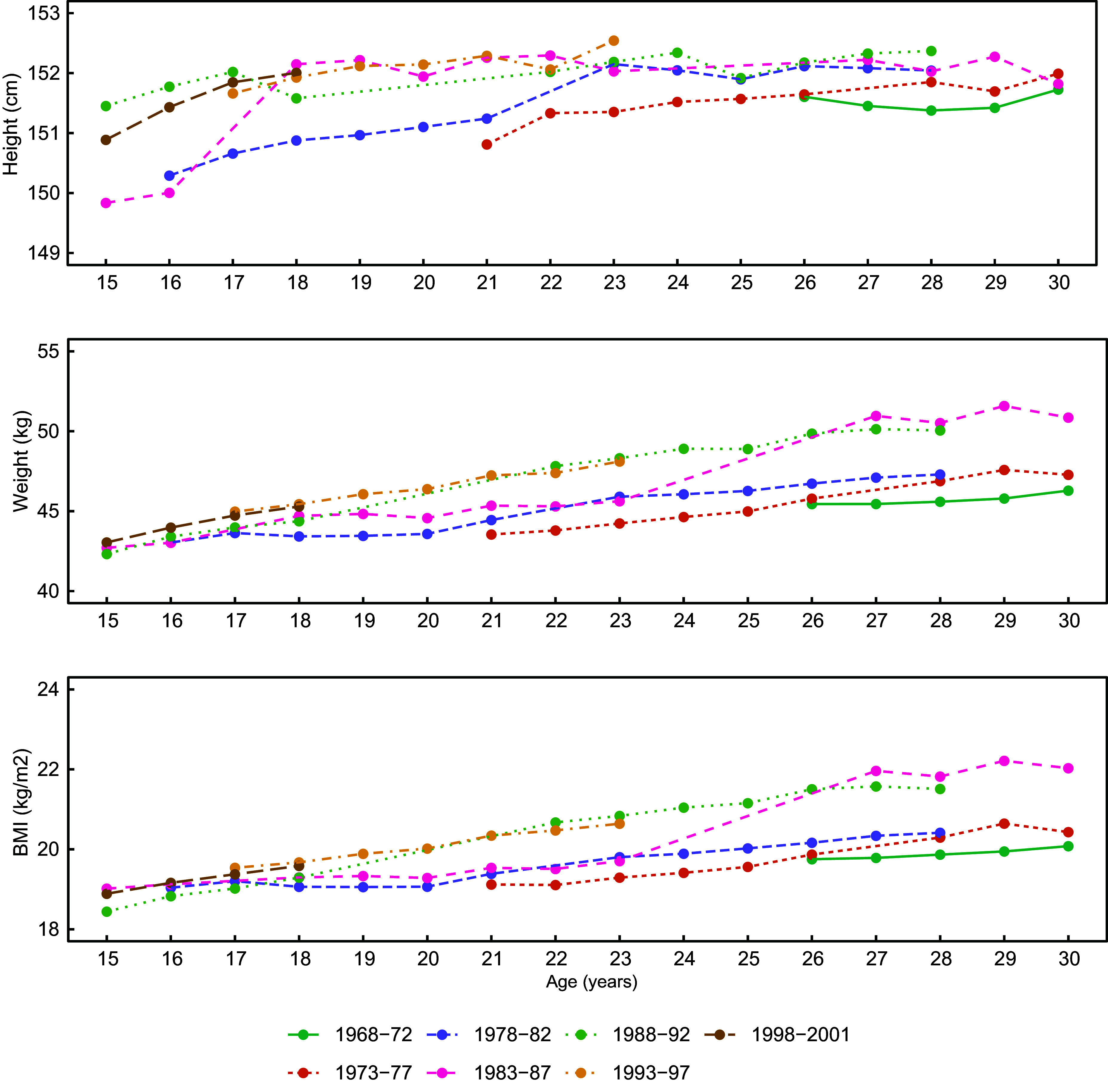
Line plot of the weighted mean of height, weight and BMI by age and birth cohort of women aged 15–30 years in the overall study sample. The mean values are not plotted where the number of women is less than 10

Figure 3 depicts age-specific line plots of mean height, weight and BMI among all women across birth cohorts. The average height of women born in the earlier cohorts (1968–1972, 1973–1977 and 1978–1982) was lower compared to the women born in the later birth cohorts at a given age. This difference was found to be more pronounced among younger women (15–20 years). The mean weight varied per birth cohort at a given age. The later birth cohorts had a higher mean weight compared to the earlier birth cohorts, especially among women older than 20 years. The mean BMI showed a similar pattern to that of weight. Overall, the women born in birth cohorts 1988–1992 and 1993–1997 were taller and heavier compared to the women born in rest of the birth cohorts across most age groups.

The mean height, weight and BMI seems to have increased with later birth cohorts among both urban and rural residing women (see online Supplemental Fig. S1). Overall, urban women were taller and heavier compared to their rural counterparts at any given age and for any birth cohort. The increase in height was steeper among urban women compared to rural women among the later cohorts. However, the mean weight and BMI of the rural women aged 15–19 years in the latest birth cohort were observed to be similar to that of urban-residing women. For example, the average BMI of urban and rural women were 19·3 kg/m^2^ (0·07) and 18·7 kg/m^2^ (0·03), respectively, at the age of 15 years and in the birth cohort 1998–2001 (see online Supplemental Fig. S1).

The mean height did not vary much across different birth cohorts at a given age among unmarried and parous ever-married women (see online Supplemental Fig. S2). However, mean height among nulliparous ever-married women did show a lot of variation across birth cohorts. The mean weight and BMI showed minimal variation across cohorts among unmarried women. Nulliparous and parous groups of ever-married women showed increasing trends in weight and BMI with later cohorts after the age of 21–22 years. Unmarried women from all birth cohorts were taller than the nulliparous and parous ever-married women belonging to the same birth cohorts at a given age (see online Supplemental Fig. S2).

### Long-term trends of height, weight and BMI by birth cohort

**Fig. 4:**
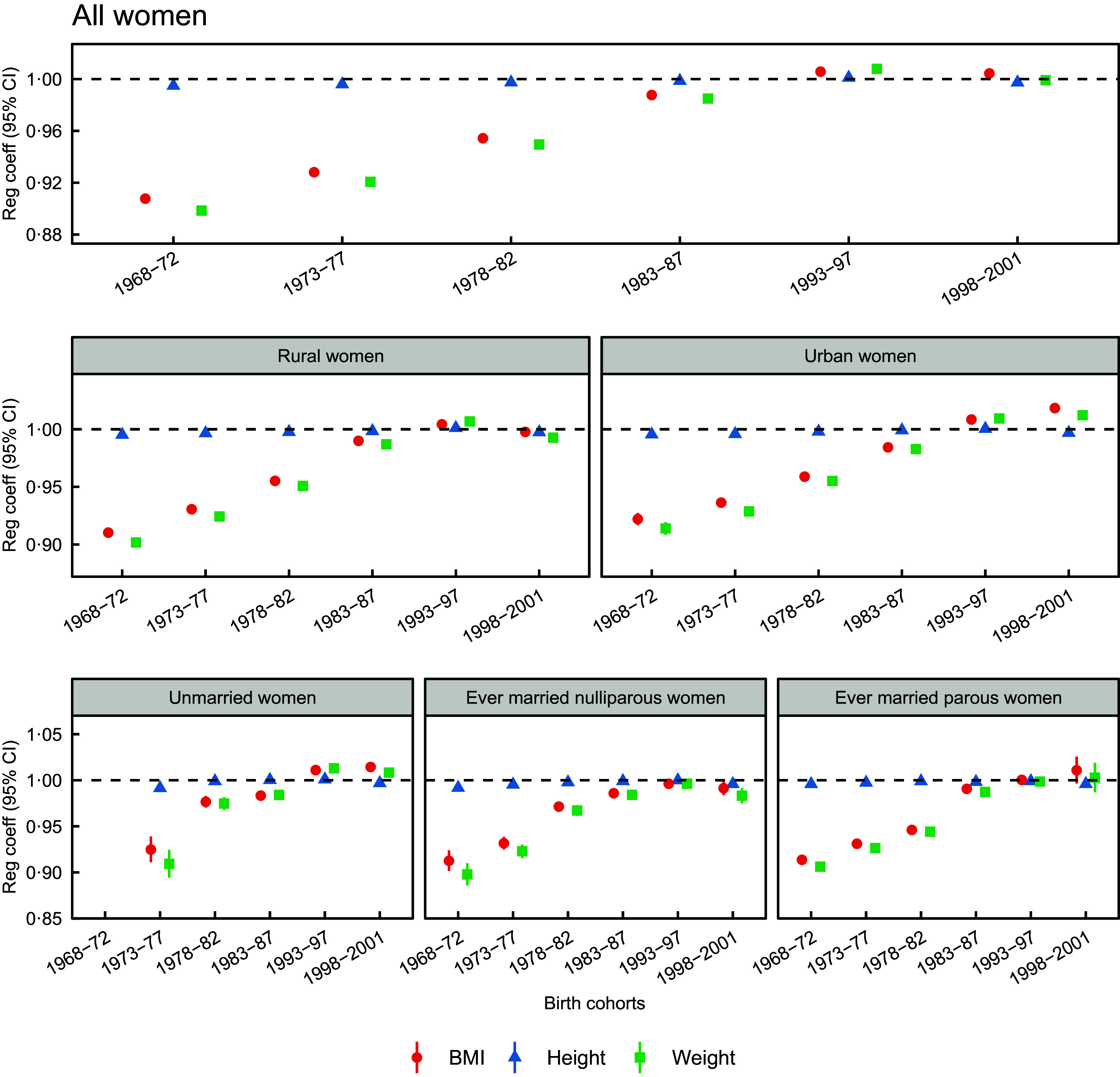
The rate of change in height, weight and BMI by birth cohorts from multivariate regression analysis in the overall study sample, by place of residence, and by marital status and parity (nulliparous and parous) among women aged 15–30 years. 1988–1992 is a reference birth cohort. In some of the places in the plots, CI of regression coefficient are too narrow to be clearly visible

Figure 4 shows the rate of change (regression coefficients) with 95 % CI across birth cohorts for height, weight and BMI for all women and stratified by place of residence and by marital status and parity. Among all women, the mean rate of change in height and the mean rate of change in weight were not identical across the birth cohorts (*P* < 0·0001). The mean rate of change in height was the lowest for the earliest birth cohort (1968–1972) (*β* = 0·995, (95 % CI 0·994, 0·996)) compared to the reference cohort (1988–1992) and it increased gradually for all the cohorts except for the latest birth cohort (1998–2001). For example, the average height of 20-year-old women born in the 1968–1972 cohort was 151·2 cm compared to 151·6 cm among those born in the 1988–1992 cohort. A parallel but much larger change was observed in the mean weight across birth cohorts. Regression coefficient of the earliest birth cohort was 0·898 (95 % CI 0·896, 0·901) compared to the reference cohort. The mean weight of the latest birth cohort did not differ significantly from the mean weight of the reference cohort (*β* = 0·999, (95 % CI 0·997, 1·002)), but the mean BMI increased (*β* = 1·004, (95 % CI 1·002, 1·006)). In summary, the rate of change in height was very low compared to the rate of change in weight, which was reflected into the rate of change in the BMI across birth cohorts among all women.

The mean height, weight and BMI differed significantly across all birth cohorts in both the urban and the rural areas. The trends in height, weight and BMI were similar to those observed among all women, except for the latest birth cohort. Rural women born in the latest birth cohort were slightly shorter and lighter and urban women were slightly shorter and heavier compared to the women in the reference cohort of their respective stratum.

The mean height, weight and BMI differed significantly across all birth cohorts in each stratum of unmarried and nulliparous and parous ever-married women. Trends in height among the three strata were consistent with the trend for height found in the overall study sample. However, when marital status and parity stratified trends in weight and BMI were compared, relatively larger increases in the regression coefficients of weight and BMI were observed among women born between 1978 and 1982 compared to the women born in the 1973–1977 birth cohort in the groups of unmarried and nulliparous ever-married women. Similar kind of increases in weight and BMI coefficients were observed one cohort later (1983–1987 *v*. 1978–1982) among parous ever-married women. The trends were very similar in the later birth cohorts (post 1988–1992) among all three strata compared to those observed among all women (Fig. 4).

In age-based subgroup analyses, similar trends in the rate of change in height, weight and BMI were observed in the two age groups (15–19 years and 20–30 years). Height increased across birth cohorts at slower pace than weight and BMI and this pattern was similar between two subgroups (results not shown). The subgroup analysis performed using the subset of currently married women showed similar results to those of the ever-married women (results not shown).

## Discussion

### Main findings

To our knowledge, this is the first study to disentangle the impact of age and birth cohorts on trends in the mean weight, height and BMI among young Indian women. The rate of change in height and weight was found to increase with succeeding birth cohorts compared with their predecessors. The increasing trend in mean weight was the key driving factor for the observed increasing trend in mean BMI across the birth cohorts. The rate of change in the mean height was similar among women residing in urban and rural India. However, the increasing trend in weight, and consequently in BMI, was steeper among urban women than among rural women. The trends in height were similar in each stratum of marital status and parity. However, when the trends in weight and BMI were compared, relatively larger increases in the regression coefficients were observed one cohort earlier among unmarried and nulliparous ever-married women than among parous ever-married women.

### Comparisons to other studies

Our analysis revealed that the mean height increased minimally in the overall study sample over the past few decades. The Non-Communicable Disease Risk Factor Collaboration analysis carried out using data of past 100 years showed that the rise in height seemed to have stopped earlier in South Asia than in East Asia^(27)^. Our findings regarding trends in height and BMI were consistent with existing cross-sectional evidence from national studies, suggesting that height has shown modest increase and overweight and obesity have increased at much faster pace in recent decades^(8,9)^. The latest birth cohort (1998–2001) showed an exceptional trend where the mean height was lower compared to the reference cohort. However, women in the latest cohort were 15–19 years of age and therefore might still have been growing at the time of the survey. In lower middle-income countries like India, people reach the adult height in their twenties^(28)^. The mean weight exhibited an increasing trend across birth cohorts except for the last one, which had similar mean weight to that of the reference cohort. Thus, the women in the latest birth cohort were shorter in height and similar in weight, which resulted in an increasing trend in BMI compared to the reference cohort. A Japanese study found that the weight of women aged 20–49 years increased with later birth cohorts, but the increment was very small, and height gradually increased as the birth cohorts became more recent^(29)^. Hence, the BMI of Japanese women increased initially and then decreased in the recent birth cohorts, which was contrary to our study. Thus, the later birth cohorts gained more weight and more BMI units at a young age than the earlier birth cohorts in our study. Many researchers globally have attempted to explain the underlying mechanisms for the trend in weight gain observed among different birth cohorts. Changing national economy leading to sedentary lifestyle, the impact of globalisation on traditional Indian diets and epidemiological transition are driving factors of rising prevalence of obesity among younger generations in India^(9,30)^.

In our study, the overall study sample comprised about 30 % of urban and 70 % of rural women in each birth cohort, reflecting the composition of urban and rural residence in Indian society. Young urban women born in the latest birth cohort were shorter, but the rate of gaining weight (and thus BMI) was higher compared to the women born in the reference cohort of 1988–1992. This finding is consistent with the work published by Jaacks *et al.* using data among young women (15–18 years) from 53 low- and middle-income countries^(31)^. Young women in urban India tend to follow urban lifestyle with an easy access to highly energy-dense food, long sitting hours in school and colleges and low physical activity levels, which can ultimately result in rapid weight gain.

The unmarried women were observed to be taller than the nulliparous or parous ever-married women at a given age across all birth cohort. This could be due to Indian men preferring wives shorter than themselves. Studies in Japan and Britain also noted a lower probability of being married as height increases among women^(32,33)^. However, a cross-sectional Indian study has reported that being taller is a positive trait for women in the Indian marriage market^(34)^. Unmarried women and nulliparous ever-married women showed a bigger increase in weight and BMI coefficients a birth cohort earlier compared to the parous ever-married women. This might be due to the fact that the earlier birth cohorts had more children on average than the later birth cohorts; resources, including food, will be shared between more people in larger families than in small families. In the Indian context, women usually eat last and least, which might lead to lower pace of weight gain.

We did not adjust for education and socio-economic status in the analysis, since the focus of our study was to evaluate the impact of birth cohorts on the trends and not to assess correlates of height, weight and BMI.

### Strengths and limitations

The study has several strengths. Firstly, three large nationally representative survey samples with high (>90 %) response rates involving women of childbearing age were used for this study. Secondly, the height and weight of each participant were objectively measured by a team of trained investigators using standard procedures. Thirdly, a robust analysis methodology was employed to model height and weight jointly, and the journey of trends in BMI was studied. Fourthly, there are no other studies focusing on height, weight and BMI through temporal dimensions of age and birth cohorts in the Indian context. Most of the previous studies have assessed only the effects of age and calendar period on these health issues. We did not adjust for education and socio-economic status in the analysis, since the focus of our study was to evaluate the impact of birth cohorts on the trends, and not to assess correlates of height, weight and BMI. To the best of our knowledge, this study is the first attempt to separately evaluate age and birth cohort effects on height, weight and BMI trends using nationally representative survey data.

The study has a few limitations. As the study was based on repeated cross-sectional surveys, it was not possible to follow the same cohort longitudinally over multiple years. The earliest survey (NFHS-2) did not include unmarried women, which prevented the study of trends in height, weight and BMI in the earliest birth cohort among unmarried women. Measurement biases were possible due to the massive nature of the study. The accuracy of self-reported birth year could be questionable, especially among illiterate participants. Due to the cross-sectional nature of the study, the analyses stratified by the place of residence might not reflect the long-term impact of the place of residence, as people may have moved from rural to urban areas, or vice versa. Therefore, it was also not possible to assess prenatal, perinatal and early-life exposures on outcomes through birth cohorts.

### Areas for future research

In the future, establishment of national longitudinal birth cohorts and the study of trends in nutritional indicators through longitudinal follow-up of the same cohorts from childhood onwards could produce more robust estimates of trends. This would enable researchers to study the impact of growth, place of residence, marital status and parity on the trends in more detail.

## Conclusion

Our results show that height and weight displayed increasing trends across birth cohorts among young Indian women aged 15–30 years. The rate of change in weight was shown to be much larger in comparison with the rate of change in height. These trends are necessarily reflected in increasing trends in BMI. Thus, birth cohort effects contribute significantly to explaining the increasing trends in BMI amongst young Indian women.

## Supplementary material

For supplementary material accompanying this paper visit https://doi.org/10.1017/S1368980021005012. click here to view supplementary material
