# Supplementary figures and images for: Birth cohort differences in height, weight and BMI among Indian women aged 15–30 years: analyses based on three cross-sectional surveys
Source: Public Health Nutr. 2021 Dec 27;25(12):3410–9. doi: 10.1017/S1368980021005012 (PMC9991631; doi:10.1017/S1368980021005012)

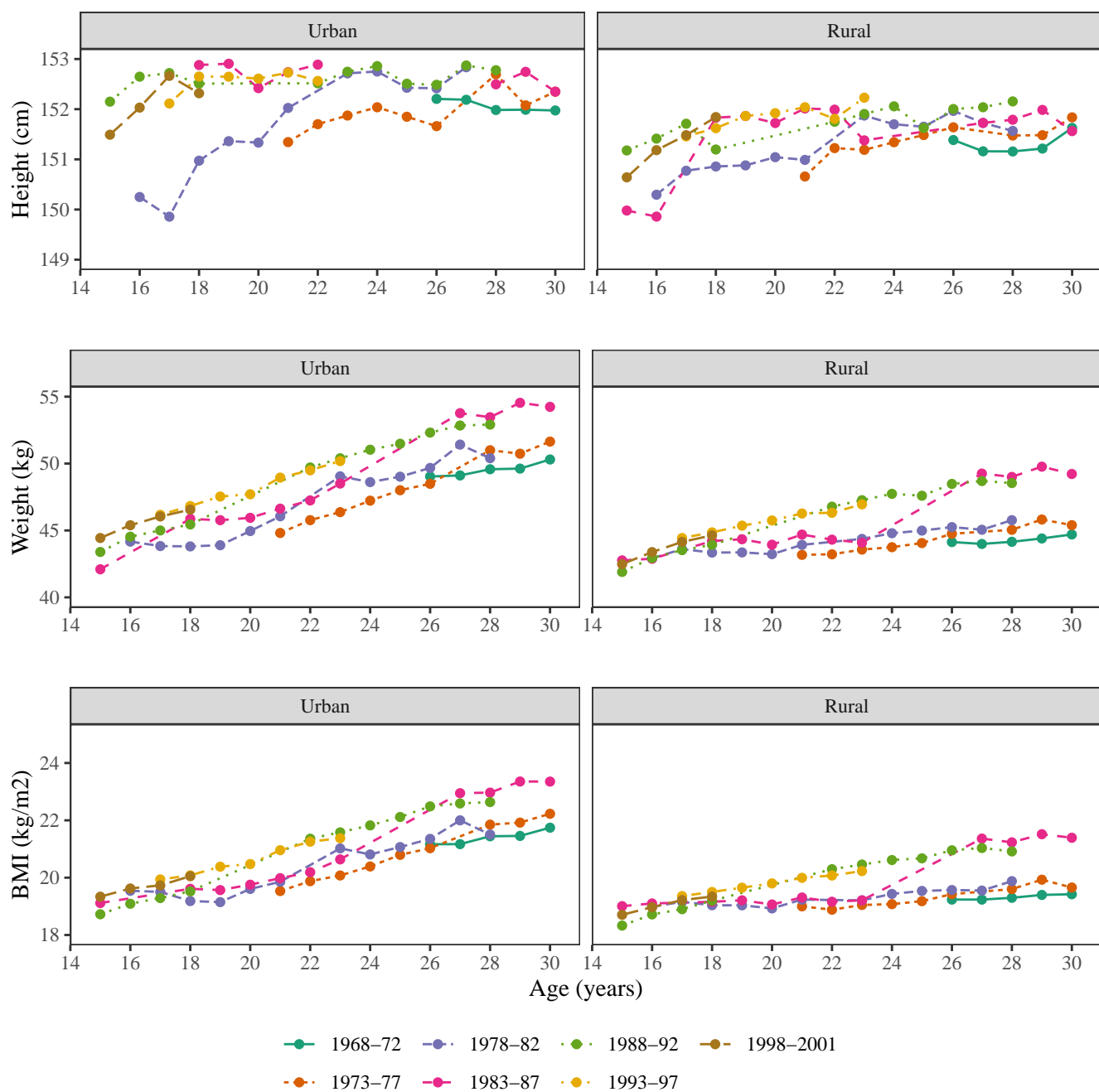

Supplement: Supplementary file 1 [file S1368980021005012sup.zip › S1368980021005012sup001.pdf]

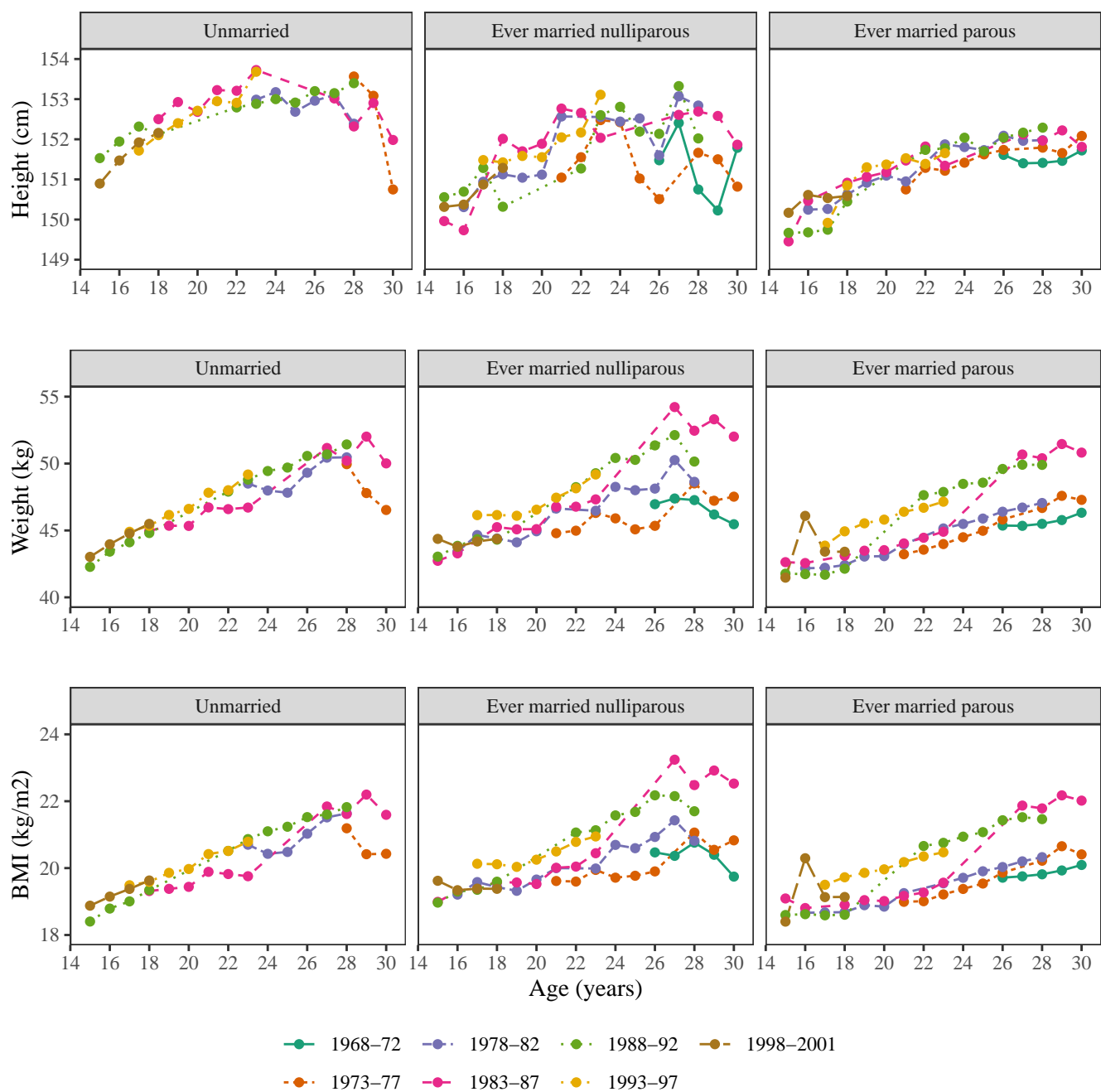

Supplement: Supplementary file 1 [file S1368980021005012sup.zip › S1368980021005012sup002.pdf]
